# KRAS G12A Identifies a High-Risk Subset in Resected Stage II–III Colorectal Cancer

**DOI:** 10.3390/cancers17223599

**Published:** 2025-11-07

**Authors:** Tomoyuki Momma, Hirokazu Okayama, Sohei Hayashishita, Daiki Yamaguchi, Ayumi Fujii, Masanori Katagata, Takuro Matsumoto, Daisuke Ujiie, Shun Chida, Zenichiro Saze, Shotaro Nakajima, Kosaku Mimura, Motonobu Saito, Wataru Sakamoto, Koji Kono

**Affiliations:** 1 Department of Gastrointestinal Tract Surgery, Fukushima Medical University School of Medicine, Fukushima 960-1295, Japan; tmomma@fmu.ac.jp (T.M.); s-h-0505@fmu.ac.jp (S.H.); daiki034@fmu.ac.jp (D.Y.); afujii61@fmu.ac.jp (A.F.); m-ktgt@fmu.ac.jp (M.K.); tak0912@fmu.ac.jp (T.M.); ujiie@fmu.ac.jp (D.U.); chida03@fmu.ac.jp (S.C.); z-saze@fmu.ac.jp (Z.S.); shotaro@fmu.ac.jp (S.N.); kmimura@fmu.ac.jp (K.M.); moto@fmu.ac.jp (M.S.); ws1024@fmu.ac.jp (W.S.); kojikono@fmu.ac.jp (K.K.); 2 Department of Multidisciplinary Treatment of Cancer and Regional Medical Support, Fukushima Medical University School of Medicine, Fukushima 960-1295, Japan; 3 Department of Blood Transfusion and Transplantation Immunology, Fukushima Medical University School of Medicine, Fukushima 960-1295, Japan; 4 Department of Cancer Genome Medicine, Fukushima Medical University Hospital, Fukushima 960-1295, Japan; 5 Department of Gastrointestinal Surgery, Aizu Medical Center, Fukushima Medical University, Fukushima 969-3492, Japan

**Keywords:** colorectal cancer, stage II–III, KRAS G12A, prognosis

## Abstract

Background: KRAS mutations are detected in ~40% of colorectal cancer (CRC), yet their prognostic value is heterogeneous across specific substitutions; the impact of uncommon variants, particularly in non-metastatic disease, remains uncertain. Methods: We evaluated the prognostic role of the relatively infrequent KRAS G12A substitution in two independent retrospective cohorts of stage II–III CRC treated with surgical resection without neoadjuvant therapy: an institutional series (FMU; n = 299) and a public dataset (AC-ICAM; n = 178). Tumors were genotyped for KRAS (and BRAF in AC-ICAM), and relapse-free survival (RFS) and overall survival (OS) were investigated. Results: KRAS G12A comprised 3.0% (FMU) and 3.4% (AC-ICAM). Across genotypes, G12A showed the highest univariable hazards compared to wild-type (WT) references for both RFS and OS in each cohort. Notably, RFS events among G12A clustered within 12 months of surgery. In multivariable Cox models, G12A remained independently associated with worse RFS and OS in each cohort, whereas non-G12A KRAS mutations did not differ significantly from the WT references. Conclusions: Across two cohorts, KRAS G12A identified a small but clinically meaningful high-risk subset of stage II–III CRC characterized by early recurrence and inferior survival. Recognition of this variant may inform postoperative risk stratification in the adjuvant setting.

## Simple Summary

Patients with stage II–III colorectal cancer (CRC) differ widely in their risk of relapse after surgery, even when they receive guideline-based adjuvant chemotherapy. Therefore, simple markers are needed to identify patients at particularly high risk after surgery. In this work, we showed that an uncommon KRAS variant, G12A, identifies a small subset of patients with stage II–III CRC who have a much higher likelihood of early recurrence and shortened survival. These findings, replicated in two independent cohorts, suggest that KRAS G12A should be recognized as a distinct high-risk marker in stage II–III disease and could be used to refine postoperative risk stratification.

## 1. Introduction

Colorectal cancer (CRC) remains a leading cause of cancer-related mortality worldwide [1,2]. For localized stage II–III disease, postoperative adjuvant chemotherapy is recommended for all stage III and selected high-risk stage II patients [2]. Nevertheless, outcomes within each stage vary widely, and only a subset of patients derive meaningful benefit from adjuvant therapy. Current clinicopathological factors, together with mismatch repair or microsatellite instability (MMR/MSI) status in stage II, provide only partial prognostic discrimination, and a considerable fraction of stage II–III patients recur after curative-intent surgery followed by adjuvant treatment [2,3,4]. Refined molecular biomarkers are therefore needed to improve postoperative risk stratification.

Oncogenic KRAS mutations are among the most prevalent driver alterations in human cancers and in CRC, constitutively activating multiple downstream cellular pathways, including those involved in cell survival and proliferation [5,6]. As the most frequently mutated oncogene across solid tumors, KRAS alterations are highly prevalent in major malignancies such as pancreatic ductal adenocarcinoma (up to 92%) and non-small cell lung cancer (30–40%), where they drive constitutive signaling and are broadly associated with adverse clinical outcomes [7,8,9]. Approximately 40% of CRCs harbor KRAS mutations, and their presence is a well-established negative predictor of benefit from epidermal growth factor receptor (EGFR)-targeted therapy in metastatic CRC (mCRC) [4,6,10]. Beyond this predictive role, the association between KRAS mutations and survival outcomes in CRC has been extensively studied. However, their prognostic impact has been modest or inconclusive, in part because not all KRAS mutations are biologically equivalent. At the codon level, codon 12 mutations have often been associated with worse overall survival (OS) and relapse-free survival (RFS) than wild-type KRAS [7,11,12,13]. At the level of specific amino acid substitutions, KRAS G12V and G12S mutations have been linked to inferior outcomes, findings most consistently reported in mCRC, whereas the prognostic effects of G12D or G13D remain controversial [7,11,12,14,15,16,17]. KRAS G12C has also been linked to unfavorable OS and progression-free survival (PFS), with implications for targeted therapeutic development for mCRC harboring this variant [5,7,12,15,18,19]. By contrast, evidence in non-metastatic stage II–III CRC following surgical resection is heterogeneous, and data on uncommon KRAS variants are especially sparse. Among these variants, KRAS G12A accounts for roughly 3–7% of KRAS-mutated CRCs [5,6,7,10]. Although KRAS G12A has been correlated with poor OS in some mCRC cohorts [20,21], its specific effect appears diluted when pooled with other variants and remains insufficiently characterized in the adjuvant setting.

In this study, we analyzed two independent retrospective cohorts (FMU and AC-ICAM), comprising 477 patients with known KRAS genotype. We aimed to elucidate the prognostic association of the KRAS G12A substitution, relative to other KRAS mutations and KRAS wild-type, with RFS and OS in patients with stage II–III CRC who underwent surgery without neoadjuvant therapy.

## 2. Materials and Methods

### 2.1. Study Population

We enrolled consecutive patients with histologically confirmed stage II–III colorectal adenocarcinoma who underwent curative surgical resection between 2014 and 2024 at Fukushima Medical University Hospital (the FMU cohort). Tumors were classified according to the Japanese Classification of Colorectal, Appendiceal, and Anal Carcinoma (JCCRC) [22]. Patients who received preoperative chemotherapy or radiotherapy were excluded. Clinical and pathological information as well as KRAS mutation testing data were obtained retrospectively from medical records. BRAF mutation data were unavailable for most tumors in this cohort. MSI testing data were also obtained from medical records, with MSI-high or MSI-positive classified as MSI-H, and MSI-low, MSI-negative, or microsatellite-stable classified as MSS. MMR status was determined by immunohistochemistry for MMR proteins (MLH1, MSH2, MSH6, and PMS2) in our previous studies [23,24,25]. Tumors exhibiting loss of at least one MMR protein were defined as dMMR, and tumors with intact MMR protein expression were classified as pMMR. In total, 299 stage II–III CRCs with KRAS mutation status were included in this study.

We also obtained a public dataset of treatment-naïve patients with CRC, complemented with curated clinical and pathological data and appropriate follow-up (the AC-ICAM cohort) [26]. For this dataset, clinical and genetic data, including KRAS/BRAF mutations and MSI status, were obtained through cBioPortal [27]. We identified 178 stage II–III CRCs with KRAS and BRAF mutational status and were eligible for RFS and OS analyses.

The study was conducted in accordance with the Declaration of Helsinki and was approved by the Institutional Review Board of Fukushima Medical University (REC2024-041).

### 2.2. Statistical Analysis

RFS was defined as the time from the date of surgery to the date of recurrence or death from any cause. OS was defined from the date of surgery to the date of death from any cause. Cumulative survival was estimated by the Kaplan–Meier method, and differences between groups were analyzed by the log-rank test. Cox proportional hazards regression was used to compute univariable and multivariable HRs and 95% CIs. Statistical analyses were performed using GraphPad Prism 9 (GraphPad Software, San Diego, CA, USA). A value of *p* < 0.05 was considered to be statistically significant.

## 3. Results

### 3.1. Clinicopathological Characteristics by KRAS Genotype in Two Independent Cohorts of Stage II–III CRC

Our investigation sought to define the prognostic significance of specific KRAS mutations in stage II–III CRC following surgical resection without prior neoadjuvant chemotherapy or radiotherapy. For this analysis, we utilized two independent cohorts: an internal institutional series (FMU cohort, n = 299) and a publicly accessible dataset obtained from cBioPortal (AC-ICAM cohort, n = 178) derived from whole-exome sequencing data [26,27]. In both cohorts, specific KRAS mutation types were identified, and for the AC-ICAM cohort, BRAF mutation status was also ascertained. Baseline clinical characteristics, including age, sex, tumor location, histological type, pathological stage, receipt of adjuvant chemotherapy, and MMR/MSI status, are summarized by genotype in Table 1 (FMU) and Table 2 (AC-ICAM). In the FMU cohort, KRAS wild-type (WT) tumors accounted for 166 cases (55.5%). Among the KRAS mutant (MT) subgroups, G12A was observed in 9 cases (3.0%), G12D in 41 cases (13.7%), G12V in 32 cases (10.7%), and G13D in 23 cases (7.7%). Similarly, in the AC-ICAM cohort, the KRAS/BRAF WT group comprised 81 cases (45.5%), while KRAS MT subgroups included G12A (n = 6; 3.4%), G12D (n = 17; 9.6%), G12V (n = 12; 6.7%), G13D (n = 8; 4.5%), with BRAF MT tumors accounting for an additional 39 cases (21.9%). Consistent with established literature, G12D was the most frequent KRAS variant across cohorts, followed by G12V and G13D, whereas G12A represented a less common subtype (~3%) [5,6,7,10]. To ensure robust statistical analysis and stable estimates for rarer alterations, KRAS mutation variants occurring at a frequency of less than 3% within each cohort were aggregated into a “KRAS MT other” category (n = 28 in FMU, and n = 15 in AC-ICAM), which encompassed variants such as G12C, G12S, A146T, and other infrequent substitutions (Supplementary Table S1).

In the FMU cohort (Table 1), distributions of age, sex, and pathological stage were broadly comparable across the various KRAS genotypes. Tumors harboring KRAS G12V were more often right-sided (53.1%) than those with KRAS WT (38.6%). Mucinous histology was relatively enriched in tumors with G12V (18.8%) and G12D (14.6%) mutations when compared to the KRAS WT group (7.2%). Overall, dMMR/MSI-H was present in 10.0%, with higher proportions found in WT (14.5%) and G13D (13.0%), and not detected in any of the G12A or G12V tumors (0.0%). In the AC-ICAM cohort (Table 2), age and stage distributions were likewise comparable among the different genotypes. BRAF MT tumors were predominantly right-sided (94.9%) and frequently exhibited MSI-H (64.1%). Conversely, KRAS/BRAF WT tumors were less often right-sided (38.3%) and rarely MSI-H (6.2%). Consistent with our observation in the FMU cohort, none of the KRAS G12A or G12V tumors in the AC-ICAM cohort displayed MSI-H (0.0%). Mucinous histology was also more prevalent in BRAF MT (41.0%), KRAS MT other (20.0%), and G12V (16.7%) tumors relative to other groups.

### 3.2. KRAS G12A Demonstrated the Worst Survival Outcomes Among KRAS Mutant Subgroups

The estimated 5-year RFS rates stratified by genotype are presented in Table 1 and Table 2, with corresponding Kaplan–Meier curves for RFS for both the FMU and AC-ICAM cohorts depicted in Supplementary Figure S1A,B. Additionally, Kaplan–Meier curves for OS for both cohorts are shown in Supplementary Figure S1C,D. Within the FMU cohort, tumors harboring KRAS G12A exhibited the lowest 5-year RFS (27.8%), whereas the rates of the remaining subgroups ranged from 58.8% to 89.0% (Table 1; Supplementary Figure S1A). Similarly, in the AC-ICAM cohort, KRAS G12A and G12V each showed a 5-year RFS of 50.0%, with other subgroups ranging from 75.0% to 81.0% (Table 2; Supplementary Figure S1C). Univariable analyses in the FMU cohort, utilizing KRAS WT as the reference group, revealed that KRAS G12A exhibited the highest HRs for both RFS (HR, 4.04; 95%CI, 1.38–9.47) and OS (HR, 6.36; 95%CI, 2.11–15.72) (Supplementary Figure S1A,B). Likewise, in the AC-ICAM cohort, with KRAS/BRAF WT as the reference, KRAS G12A demonstrated the highest hazards for both RFS (HR, 4.16; 95%CI, 0.96–12.64) and OS (HR, 3.54; 95%CI, 1.03–9.33) (Supplementary Figure S1C,D). While KRAS G12V was also significantly associated with inferior RFS in the AC-ICAM cohort (HR, 3.19; 95%CI, 1.14–7.86), this pattern was not observed in the FMU cohort (Supplementary Figure S1A–D). A striking and consistent observation across both cohorts was the early accumulation of RFS events for KRAS G12A predominantly within 12 months of surgery: 3 out of 4 events (75%) in FMU cohort and all 3 out of 3 events (100%) in AC-ICAM cohort (Supplementary Figure S1A,C).

### 3.3. Independent Prognostic Significance of KRAS G12A in Stage II–III CRC

**Figure 1:**
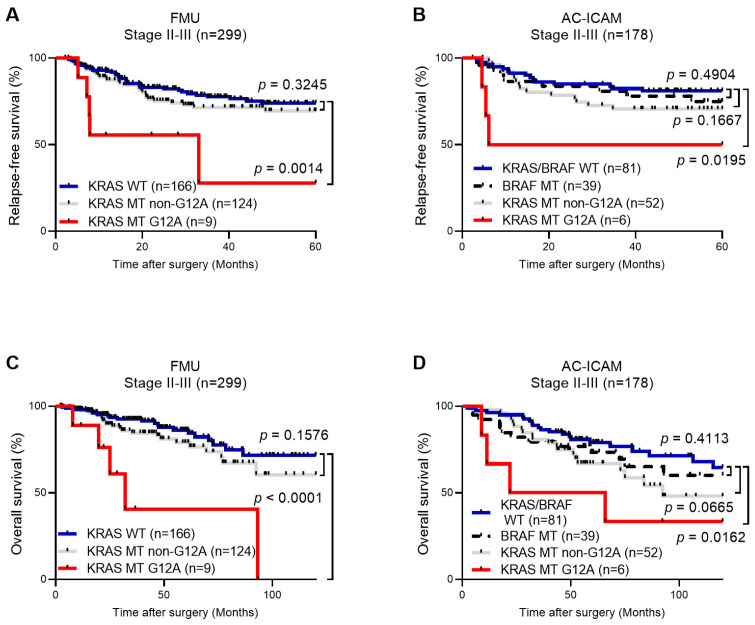
Kaplan–Meier curves for relapse-free survival (**A**,**B**) and overall survival (**C**,**D**) in stage II–III colorectal cancer. Tumors are classified according to KRAS wild-type (WT), KRAS mutant (MT) non-G12A, and KRAS MT G12A in the FMU cohort (**A**,**C**), and KRAS/BRAF WT, BRAF MT, KRAS MT non-G12A, and KRAS MT G12A, in the AC-ICAM cohort (**B**,**D**). Log-rank *p* values are indicated.

Given the consistently adverse outcomes observed for KRAS G12A across genotype-defined analyses, we next adopted a clinically pragmatic stratification that dichotomized KRAS MT tumors into G12A and non-G12A (comprising KRAS MT G12D, G12V, G13D, and KRAS MT other). This yielded three analysis groups in the FMU cohort, including KRAS WT (n = 166), KRAS MT non-G12A (n = 124), and KRAS MT G12A (n = 9), and four groups in the AC-ICAM cohort, including KRAS/BRAF WT (n = 81), BRAF MT (n = 39), KRAS MT non-G12A (n = 52), and KRAS MT G12A (n = 6). For both cohorts, Kaplan–Meier curves for RFS and OS are shown in Figure 1A–D, and univariable and multivariable Cox analyses for RFS and OS are summarized in Table 3. In these analyses, KRAS WT in the FMU cohort and KRAS/BRAF WT in the AC-ICAM cohort served as the reference groups. Across cohorts, KRAS G12A consistently demonstrated the poorest RFS and OS, with a clear and statistically significant separation of Kaplan–Meier curves from the reference groups (Figure 1A–D; log-rank *p*-values are shown in the figure). By contrast, KRAS MT non-G12A and BRAF MT groups did not significantly differ from their respective WT references for either RFS or OS (Figure 1A–D). In multivariable Cox models adjusted for established clinicopathological factors including age, sex, tumor location, histological type, pathological stage, receipt of adjuvant chemotherapy, and MMR/MSI status, KRAS G12A remained an independent adverse prognostic factor for both endpoints, in both the FMU cohort (RFS: adjusted HR, 5.23; 95%CI, 1.50–14.11; *p* = 0.003; OS: adjusted HR, 10.64; 95%CI, 2.39–34.00; *p* < 0.001) and in the AC-ICAM cohort (RFS: adjusted HR, 5.13; 95%CI, 1.15–16.44; *p* = 0.013; OS: adjusted HR, 3.83; 95%CI, 1.08–10.61; *p* = 0.018). In contrast, the non-G12A category remained non-significant after adjustment in both cohorts.

## 4. Discussion

In the present study, analyses of two independent cohorts revealed that the KRAS G12A substitution can identify a small but clinically actionable subset within stage II–III CRC associated with poor survival outcomes. Indeed, among the broader KRAS mutational landscape, KRAS G12A has been known to occur at low frequencies in CRC, comprising approximately 3–7% [5,6,7,10], in line with our observation: 3.0% in the FMU cohort and 3.4% in the AC-ICAM cohort. We found that G12A tumors were associated with the poorest RFS and OS among KRAS genotypes, and these associations remained significant after adjustment for standard clinicopathological factors, adjuvant chemotherapy, and MMR/MSI status. A particularly striking and clinically relevant observation was that KRAS G12A tumors recurred early, with the majority of RFS events occurring within 12 months of surgery. Collectively, these findings support KRAS G12A as a postoperative prognostic marker in stage II–III CRC. In the current clinical practice, KRAS mutational status, including G12A, can be readily detectable, particularly in the metastatic setting [4,6]. Our data suggest that the simple identification of KRAS G12A at routine genotyping in the adjuvant setting could offer actionable risk stratification beyond current clinicopathological variables as well as MMR/MSI status. Therefore, patients with stage II–III CRC harboring KRAS G12A, representing a small but high-risk subset, may benefit from more potent adjuvant chemotherapy regimens, combined with more intensive postoperative surveillance. While direct inhibitors for KRAS G12A are not yet available, unlike those for G12C [5,6,7,19], the ongoing revolution in KRAS drug discovery, including pan-KRAS inhibitors, might offer therapeutic possibilities that could be selectively applied to this high-risk group.

The predictive utility of overall KRAS mutations for guiding anti-EGFR therapy in mCRC is well-established. Moreover, the prognostic and predictive role of specific KRAS substitutions or codon-specific KRAS mutations has been extensively investigated [7]. For instance, G12V, G12C, and G12S have been linked to inferior outcomes, whereas others, such as G12D and G13D, show variable effects [7,11,12,14,15,16,17,18]. KRAS G12 mutations were predictive of reduced OS benefit from trifluridine/tipiracil chemotherapy in mCRC [28]. KRAS A146 mutations were associated with inferior survival in patients with liver metastasis [29]. However, these observations in prior studies have largely relied on relatively heterogeneous populations of advanced or recurrent metastatic disease, in which patients were already exposed to diverse systemic treatments. Evidence in non-metastatic, surgically treated populations has been inconclusive, where results across numerous studies often appear inconsistent. The present study addressed this critical gap by analyzing a relatively uniform population of surgically resected stage II–III patients without neoadjuvant therapy and explicitly separating G12A from other KRAS mutation variants. Therefore, by minimizing confounding factors commonly seen in the metastatic setting, our work provides a direct assessment of the intrinsic prognostic impact of KRAS G12A in earlier stage disease. Furthermore, the consistent observations in two independent cohorts significantly enhance the robustness of our conclusions. Our identification of the distinct aggressive phenotype of KRAS G12A in resected stage II–III CRC is also supported by previous studies focusing on mCRC. Fiala et al. reported that KRAS G12V and G12A mutations were associated with inferior PFS and OS in patients with mCRC treated with bevacizumab [21]. Similarly, Tonello et al. identified a subgroup of KRAS mutations (KRASMUT2), which included G12A, correlating with significantly worse OS in patients with peritoneal metastases [30]. Peeters et al. conducted a pooled analysis in mCRC and found G12A to be associated with a negative predictive effect on OS with panitumumab [20]. Jones et al. reported that G12C and G12V were predictive of poor OS in patients with recurrent and metastatic disease, and G12A also showed a trend toward poor OS [15]. Therefore, our study and these previous studies consistently indicate the deleterious effects of G12A on prognosis in both the non-metastatic and metastatic settings. Moreover, our observation that KRAS G12A tumors showed an early recurrence pattern, even in the absence of overt metastatic disease, suggests a unique tumor biology of G12A driving a particularly aggressive behavior from its early stages, rather than poor responsiveness to specific target therapies in advanced settings. Although the biological basis for the G12A phenotype remains to be clarified and preclinical data specific to G12A are limited compared with other variants [5,7], further mechanistic work is crucial to dissect the specific molecular mechanisms by which KRAS G12A confers this aggressive phenotype.

Across the pan-cancer landscape, KRAS G12A is also relatively uncommon, accounting for approximately 4% of all KRAS mutations detected, a frequency observed consistently in pan-tumor tissue data and liquid genotyping assays [5,8]. However, the frequency is highly dependent on the tumor type. For example, in pancreatic ductal adenocarcinoma, which is highly dependent on KRAS signaling, G12A remains a notably rare variant, representing roughly 0.8% of KRAS-mutant tumors, whereas in non–small cell lung cancers, G12A accounts for a more substantial proportion, reported at approximately 10% of KRAS-mutant cases [5,7,8,31]. Because of this low prevalence, comprehensive translational or biological studies examining the behavior of G12A are limited, and its independent prognostic and predictive significance beyond CRC remains largely undefined.

Despite the strengths of our study, including the use of two independent cohorts and a focused patient population, several limitations remain. Its retrospective nature may introduce inherent biases, possibly due to variations in adjuvant therapy regimens, follow-up intensity and molecular profiling platforms. Since the rarity of G12A led to relatively small subgroup sizes with a limited number of survival events, these results should be interpreted with caution despite reproducible findings in two different cohorts. Future studies should validate these findings using larger, prospective cohorts of stage II–III CRC, and explore mechanistic underpinnings by which KRAS G12A confers an aggressive phenotype.

## 5. Conclusions

Our study provides compelling evidence that KRAS G12A mutation is an independent and clinically useful adverse prognostic factor, identifying a distinct high-risk subset of resected stage II–III CRC patients characterized by early recurrence and inferior survival. Recognizing G12A as a prognostic biomarker may improve postoperative risk stratification to guide personalized management of adjuvant chemotherapy and surveillance.

## Figures and Tables

**Table 1:** Characteristics of stage II–III colorectal cancer according to KRAS mutations in the FMU cohort.

		Total	KRAS WT	KRAS MT G12A	KRAS MT G12D	KRAS MT G12V	KRAS MT G13D	KRAS MT Other
		n = 299	n = 166 (55.5%)	n = 9 (3.0%)	n = 41 (13.7%)	n = 32 (10.7%)	n = 23 (7.7%)	n = 28 (9.4%)
Age								
	Mean ± SD	70.1 ± 11.1	69.4 ± 11.4	67.0 ± 13.8	72.2 ± 9.6	72.6 ± 10.8	69.0 ± 9.8	70.6 ± 12.0
Sex								
	Male	175 (58.5%)	97 (58.4%)	4 (44.4%)	23 (56.1%)	18 (56.3%)	16 (69.6%)	17 (60.7%)
	Female	124 (41.5%)	69 (41.6%)	5 (55.6%)	18 (43.9%)	14 (43.8%)	7 (30.4%)	11 (39.3%)
Tumor location								
	Right	118 (39.5%)	64 (38.6%)	3 (33.3%)	14 (34.1%)	17 (53.1%)	11 (47.8%)	9 (32.1%)
	Left	181 (60.5%)	102 (61.4%)	6 (66.7%)	27 (65.9%)	15 (46.9%)	12 (52.2%)	19 (67.9%)
Histological type								
	Well/Moderately differentiated	263 (88.0%)	148 (89.2%)	8 (88.9%)	35 (85.4%)	26 (81.3%)	20 (87.0%)	26 (92.9%)
	Poorly differentiated	7 (2.3%)	5 (3.0%)	0 (0.0%)	0 (0.0%)	0 (0.0%)	1 (4.3%)	1 (3.6%)
	Mucinous	27 (9.0%)	12 (7.2%)	1 (11.1%)	6 (14.6%)	6 (18.8%)	1 (4.3%)	1 (3.6%)
	Signet-ring cell/Undifferentiated	2 (0.7%)	1 (0.6%)	0 (0.0%)	0 (0.0%)	0 (0.0%)	1 (4.3%)	0 (0.0%)
pathological stage								
	stage II	141 (47.2%)	76 (45.8%)	5 (55.6%)	23 (56.1%)	15 (46.9%)	8 (34.8%)	14 (50.0%)
	stage III	158 (52.8%)	90 (54.2%)	4 (44.4%)	18 (43.9%)	17 (53.1%)	15 (65.2%)	14 (50.0%)
Adjuvant chemotherapy								
	No	144 (48.2%)	77 (46.4%)	4 (44.4%)	22 (53.7%)	17 (53.1%)	8 (34.8%)	16 (57.1%)
	Yes	155 (51.8%)	89 (53.6%)	5 (55.6%)	19 (46.3%)	15 (46.9%)	15 (65.2%)	12 (42.9%)
MMR/MSI status								
	dMMR/MSI-H	30 (10.0%)	24 (14.5%)	0 (0.0%)	3 (7.3%)	0 (0.0%)	3 (13.0%)	0 (0.0%)
	pMMR/MSS	257 (86.0%)	136 (81.9%)	7 (77.8%)	37 (90.2%)	31 (96.9%)	19 (82.6%)	27 (96.4%)
	undetermined	12 (4.0%)	6 (3.6%)	2 (22.2%)	1 (2.4%)	1 (3.1%)	1 (4.3%)	1 (3.6%)
5-year RFS rate								
	%	70.9%	74.0%	27.8%	58.8%	64.4%	71.5%	89.0%

WT, wild-type; MT, mutant; dMMR/MSI-H, deficient mismatch-repair/microsatellite instability-High; pMMR/MSS, proficient mismatch-repair/microsatellite stable; RFS, relapse-free survival.

**Table 2:** Characteristics of stage II–III colorectal cancer according to KRAS and BRAF mutations in the AC-ICAM cohort.

		Total	KRAS/BRAF WT	KRAS MT G12A	KRAS MT G12D	KRAS MT G12V	KRAS MT G13D	KRAS MT other	BRAF MT
		n = 178	n = 81 (45.5%)	n = 6 (3.4%)	n = 17 (9.6%)	n = 12 (6.7%)	n = 8 (4.5%)	n = 15 (8.4%)	n = 39 (21.9%)
Age									
	Mean ± SD	68 ± 11.7	66.1 ± 12.5	66.3 ± 16.4	66.9 ± 14.4	71.0 ± 8.5	65.9 ± 12.1	69.9 ± 8.3	71.6 ± 8.9
Sex									
	Male	94 (52.8%)	49 (60.5%)	4 (66.7%)	6 (35.3%)	8 (66.7%)	5 (62.5%)	10 (66.7%)	12 (30.8%)
	Female	84 (47.2%)	32 (39.5%)	2 (33.3%)	11 (64.7%)	4 (33.3%)	3 (37.5%)	5 (33.3%)	27 (69.2%)
Tumor location									
	Right	103 (57.9%)	31 (38.3%)	3 (50.0%)	13 (76.5%)	6 (50.0%)	4 (50.0%)	9 (60.0%)	37 (94.9%)
	Left	75 (42.1%)	50 (61.7%)	3 (50.0%)	4 (23.5%)	6 (50.0%)	4 (50.0%)	6 (40.0%)	2 (5.1%)
**Histological type**									
	Adenocarcinoma	143 (80.3%)	72 (88.9%)	6 (100.0%)	14 (82.4%)	10 (83.3%)	7 (87.5%)	11 (73.3%)	23 (59.0%)
	Mucinous adenocarcinoma	31 (17.4%)	8 (9.9%)	0 (0%)	2 (11.8%)	2 (16.7%)	0 (0.0%)	3 (20.0%)	16 (41.0%)
	Signet-ring cell/Cribriform carcinoma	4 (2.2%)	1 (1.2%)	0 (0%)	1 (5.9%)	0 (0.0%)	1 (12.5%)	1 (6.7%)	0 (0.0%)
pathological stage									
	stage II	97 (54.5%)	41 (50.6%)	4 (66.7%)	7 (41.2%)	6 (50.0%)	5 (62.5%)	7 (46.7%)	27 (69.2%)
	stage III	81 (45.5%)	40 (49.4%)	2 (33.3%)	10 (58.8%)	6 (50.0%)	3 (37.5%)	8 (53.3%)	12 (30.8%)
Adjuvant chemotherapy									
	No	119 (66.9%)	50 (61.7%)	4 (66.7%)	11 (64.7%)	7 (58.3%)	6 (75.0%)	9 (60.0%)	32 (82.1%)
	Yes	59 (33.1%)	31 (38.3%)	2 (33.3%)	6 (35.3%)	5 (41.7%)	2 (25.0%)	6 (40.0%)	7 (17.9%)
MSI status									
	MSI-H	33 (18.5%)	5 (6.2%)	0 (0.0%)	1 (5.9%)	0 (0.0%)	2 (25.0%)	0 (0.0%)	25 (64.1%)
	MSS	145 (81.5%)	76 (93.8%)	6 (100.0%)	16 (94.1%)	12 (100.0%)	6 (75.0%)	15 (100.0%)	14 (35.9%)
5-year RFS rate									
	%	75.4%	81.0%	50.0%	75.0%	50.0%	75.0%	80.0%	74.9%

WT, wild-type; MT, mutant; MSI-H, microsatellite instability-High; MSS, microsatellite stable; RFS, relapse-free survival.

**Table 3:** Cox proportional hazards models for relapse-free survival (RFS) and overall survival (OS) in stage II-III colorectal cancer.

	RFS				OS			
	Univariable		Multivariable		Univariable		Multivariable	
	HR (95% CI)	*p*	HR (95% CI)	*p*	HR (95% CI)	*p*	HR (95% CI)	*p*
FMU cohort (n = 299)								
KRAS WT	1 (Referent)		1 (Referent)		1 (Referent)		1 (Referent)	
KRAS MT G12A	4.04 (1.38–9.46)	0.004	5.23 (1.50–14.11)	0.003	6.37 (2.11–15.74)	<0.001	10.64 (2.39–34.00)	<0.001
KRAS MT non-G12A	1.28 (0.78–2.08)	0.324	1.09 (0.66–1.80)	0.737	1.54 (0.85–2.81)	0.152	1.35 (0.73–2.51)	0.344
AC-ICAM cohort (n = 178)								
KRAS/BRAF WT	1 (Referent)		1 (Referent)		1 (Referent)		1 (Referent)	
KRAS MT G12A	4.16 (0.96–12.64)	0.024	5.13 (1.15–16.44)	0.013	3.54 (1.03–9.33)	0.021	3.83 (1.08–10.61)	0.018
KRAS MT non-G12A	1.64 (0.80–3.38)	0.176	2.05 (0.94–4.46)	0.069	1.73 (0.93–3.21)	0.081	1.80 (0.93–3.47)	0.079
BRAF MT	1.35 (0.57–3.02)	0.481	2.68 (0.83–8.03)	0.087	1.33 (0.65–2.63)	0.416	2.60 (1.02–6.23)	0.037

All multivariable models were adjusted for age, sex, tumor location, histological type, pathological stage, receipt of adjuvant chemotherapy, mismatch repair/microsatellite instability status. HR, hazard ratio; CI, confidence interval; WT, wild-type; MT, mutant.

## Data Availability

The AC-ICAM dataset used in this study is available from cBioPortal (https://www.cbioportal.org/, accessed on 28 August 2025).

## Supplementary Materials

The following supporting information can be downloaded at: https://www.mdpi.com/article/10.3390/cancers17223599/s1, Figure S1: Kaplan–Meier curves for relapse-free and overall survival by genotype; Table S1: Uncommon KRAS mutation frequencies.

## Abbreviations

The following abbreviations are used in this manuscript:

CRC	colorectal cancer
mCRC	metastatic CRC
EGFR	epidermal growth factor receptor
FMU	Fukushima Medical University
RFS	relapse-free survival
OS	overall survival
PFS	progression-free survival
MMR/MSI	mismatch repair or microsatellite instability
dMMR/MSI-H	deficient mismatch repair or microsatellite instability-High
HR	hazard ratio
CI	confidence interval
WT	wild-type
MT	mutant
