# Supplementary material for: KRAS G12A Identifies a High-Risk Subset in Resected Stage II–III Colorectal Cancer
Source: Cancers (Basel). 2025 Nov 7;17(22):3599. doi: 10.3390/cancers17223599 (PMC12651688; doi:10.3390/cancers17223599)
Supplement: Supplementary file 1 [file cancers-17-03599-s001.zip › cancers-3952176-supplementary.pdf]

Supplementary Table S1. Uncommon KRAS mutation frequencies (<3.0%).

	KRAS MT other in FMU cohort n=28 (9.4%)	KRAS MT other in AC-ICAM cohort n=15 (8.4%)
G12C	7 (2.3%)	2 (1.1%)
G12R	1 (0.3%)	1 (0.6%)
G12S	4 (1.3%)	2 (1.1%)
G13C	1 (0.3%)	0 (0.0%)
A59T	0 (0.0%)	2 (1.1%)
Q61H	1 (0.3%)	2 (1.1%)
Q61R	1 (0.3%)	2 (1.1%)
K117N	5 (1.7%)	0 (0.0%)
A146P	1 (0.3%)	0 (0.0%)
A146T	5 (1.7%)	2 (1.1%)
A146V	0 (0.0%)	1 (0.6%)
Double	2 (0.7%)	1 (0.6%)

Supplementary Figure S1

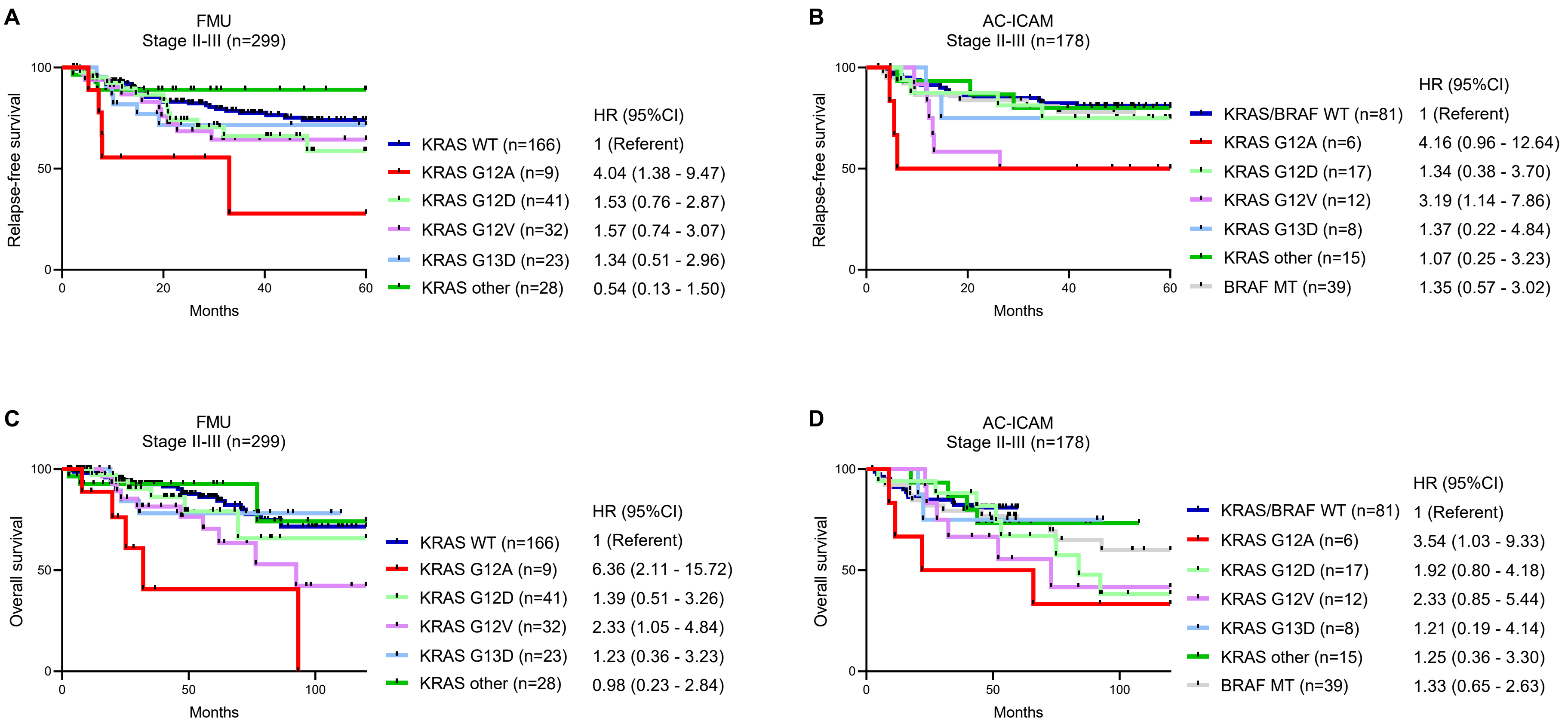

Supplementary Figure S1. Kaplan–Meier curves for relapse-free survival (A,B) and overall survival (C,D) by genotype in stage II–III colorectal cancer for the FMU (A,C) and AC-ICAM (B,D) cohorts; corresponding univariable hazard ratios (HR) with 95% confidence intervals (CI) versus KRAS wild-type (FMU) or KRAS/BRAF wild-type (AC-ICAM) are also shown.
